# Estimation of Spleen Volume Using MRI Segmentation: Would One Slice Be Enough?

**DOI:** 10.7759/cureus.32165

**Published:** 2022-12-03

**Authors:** Waleed Mohammed, Nizar Ismail, Mogahid Zidan, Ala Elgyoum, Hussein Hassan, Osman Abdelrahman

**Affiliations:** 1 Diagnostic Imaging, The National Ribat University, Khartoum, SDN; 2 Trauma and Orthopaedics, Royal Cornwall Hospital, Truro, GBR; 3 Trauma and Orthopaedics, Buckinghamshire Healthcare NHS Trust, Aylesbury, GBR; 4 Medicine, The National Ribat University, Khartoum, SDN; 5 Radiology, The National Ribat University, Khartoum, SDN; 6 Radiology, Karary University, Khartoum, SDN; 7 Anatomy, University of Medical Sciences and Technology, Khartoum, SDN

**Keywords:** volume, splenomegaly, spleen, segmentation, mri, cavalieri

## Abstract

This study aimed to establish spleen volume in adult Sudanese using manual segmentation and the Cavalieri method with magnetic resonance images (MRI). A total of 345 abdominal MRI scans for adults were retrospectively studied for patients who underwent abdomen MRI between September to October 2016. The data were collected from two scanning centers in Khartoum, Sudan. For each series of sectional images, the spleen was manually segmented, and the volume was calculated using matrix laboratory (MATLAB) code. Furthermore, abdominal circumferences and diameters, in addition to L1 body dimension and spinal canal, were measured.

The mean splenic volume in our study was 187.2 cm^3^, without a significant change with age (P = 0.269). The average volume in males was 223.5 cm^3^ while in females it was 170.27 cm^3^. The average coefficient error (CE) was 0.029. A positive significant correlation was found between the volume and spleen length (beta standardized coefficient = 0.781, P < 0.05). The mean length was 9.18 cm. Additionally, our data showed an insignificant correlation between the volume and the other measurements, except for the abdomen transverse diameter (beta standardized coefficient = 0.267, P < 0.05).

The results of our study coincided with previous studies in the normal range of spleen volume and the strong correlation with length, regardless of the irregular shape of the spleen. In addition, manual segmentation was a reliable method to measure spleen volume objectively. However, our single-slice measurements were insufficient.

## Introduction

The size, shape, and position of the spleen vary hugely among individuals. In addition, the spleen size changes might be physiological or transient. For example, the volume of the spleen reduces for a short time during a breath hold [1].

Although spleen diseases are uncommon, the spleen volume (SV) is affected by several hematological and immunological disorders such as lymphoma, rheumatoid arthritis, and Gaucher's disease. It plays an important role amid liver volume in monitoring and predicting the outcome of chronic liver disease and fibrosis [2]. Moreover, along with the lung volume, the SV is used to estimate the performance of apneic divers [3].

In clinical practice, the spleen is palpable only when it is two to three times its reference size, a clinical condition known as splenomegaly. Therefore, medical imaging modalities are used to give more accurate measurements and provide a normal range of SV. Regardless of the reliability of each, almost all imaging modalities, including plain film X-rays, could be used for spleen size estimation. However, ultrasonography (US), computed tomography (CT), and magnetic resonance imaging (MRI) are most commonly used for measuring the volume of intra-abdominal organs. Both CT and MRI overcome the limitations present in an US, for instance, ribs and gases overlapping and low signal-to-noise ratio. That is because of the CT and MRI's higher spatial and contrast resolutions and their superior ability of three-dimensional rendering [4,5].

Various methods have been combined with sectional imaging modalities to estimate the spleen size in vivo. The most common practice is the one-dimensional (1D) measure of spleen length with an US. Another technique uses three (1D) measurements to calculate the splenic index, which interprets spleen length, width, and thickness. However, these methods are still insufficient to derive accurate information about a three-dimensional (3D) irregular object such as the spleen [5,6,7].

The Cavalieri method is one of the volumetric techniques that estimate the volume of an organ regardless of its shape from a set of two-dimensional (2D) slices through the organ [1]. In this method, slices or sections should be parallel, separated by a known distance, and begin randomly within the organ. Such criteria are fulfilled by MRI and CT. In addition, the precision of the Cavalieri principle estimate can be calculated by figuring out the coefficient error (CE). A CE equal to or less than 0.05 is acceptable [8].

Many studies reported the range of normal SV as another method of determining individuals' spleen size. The normal range is wide, and unless the SV of a subject was measured previously, relatively small changes cannot be determined [9,10,11]. For this reason, various trends searched the correlation of SV with age, height, weight, and other anthropometric measures to predict a specific individual's splenic volume. Several studies reported a moderate correlation of SV with the height and weight of the person [3,5,10]. Moreover, Geraghty et al. found a correlation between weight and height, with lumber vertebra measurements and abdominal circumference on axial CT images at level L1 [12].

In this study, we aimed to establish reference values of SV in the adult Sudanese population using manual segmentation and the Cavalieri methods along with MRI. In addition, we also aimed to determine the association of the SV and splenic length, dimensions of the slice of the abdomen, and vertebral body at the level of the first lumbar vertebra.

## Materials and methods

The MRI abdomen data of 345 adult patients (110 male and 235 females) who underwent scanning from September to October 2016 were retrospectively collected from Al-Zytouna Specialist Hospital and El-Nilein Medical Diagnostic Centre in Khartoum, Sudan. The abdominal region was scanned for various clinical conditions. Although the scanning protocols differed from one manufacturer to another, all patients were scanned with the free-breathing technique. For each patient, images were verified to be satisfactory; the patient's age was above 18 years at the time of the scan, the spleen appearance and report were normal, and the MRI slices covered the whole spleen. Magnetic resonance imaging examination was done by using two MRI machines: Toshiba 1.5 Tesla MRI unit (Toshiba Medical Systems, Tokyo, Japan) and Siemens 0.35 Tesla MRI unit (Siemens Medical Systems, Munich, Germany). Ethical considerations were followed and consent was obtained as per institutional policies.

Measurements for the manual segmentation of the spleen and images

For each subject, the spleen was segmented manually, and the number of pixels of each slice was counted using intensity threshold segmentation. The digital imaging and communications in medicine (DICOM) meta-data were used to extract voxel dimensions and slice thickness and spacing. The spleen slice area was calculated by multiplying the pixel dimension and the number of spleen pixels for each slice. Subsequently, the volume was calculated by multiplying slice spacing (slice thickness and gap between slices) and the sum of areas of all slices. The areas of the L1 body and spinal canal were calculated similarly. However, abdominal circumference measurement had a slightly similar but different approach. The image was manually processed and then subjected to an open-area morphological function, which results in the circumference being countered by a one-pixel width line of pixels. Then, the number of pixels was multiplied by the pixel’s length. Then all other dimensions were painted, segmented, and calculated in pixel length. All measurement steps were conducted twice by two independent radiographers, and only the average was presented as the result. The CE was estimated using the Cruz-Orive equation, as described by Robots et al. as the best formula for irregularly shaped objects [13]. 

Statistical analysis

The data were analyzed using Statistical Package for the Social Sciences (SPSS) version 20 (IBM Corp., Armonk, NY, USA) and Microsoft Excel 2007 (Microsoft Corp., Redmond, WA, USA). A descriptive summary of the data was obtained. Moreover, associations between independent variables and dependent variables (SV) were investigated using the ANOVA test.

## Results

Out of the 345 MRI reports obtained from Al-Zytouna Specialist Hospital and El-Nilein Medical Diagnostic Centre in Khartoum, Sudan, 110 (31.9%) belonged to females, while 235 (68.1%) belonged to males (Table 1).

**Table 1:** Gender of patients who underwent an MRI abdomen between September and October 2016

Gender	Frequency	Percentage
Male	110	31.9
Female	235	68.1
Total	345	100.0

The age ranges of the patients were as follows: 125 (36.2%) patients were aged 20 to 44 years, 127 patients (36.8%) were of 45 to 64 years, 89 patients (25.8%) were of 65 to 84 years, and four patients (1.2%) were aged more than 84 years (Table 2).

**Table 2:** Age of patients who underwent an MRI abdomen between September and October 2016

Age	Frequency	Percentage
20-44	125	36.2
45-64	127	36.8
65-84	89	25.8
>84	4	1.2
Total	345	100.0

Out of the 345 SVs measured, 310 were classified as normal, while 35 were classified as high spleen volumes (Table 3).

**Table 3:** Spleen volume among patients who underwent an MRI abdomen between September and October 2016

Spleen volume	Frequency	Percentage
Normal	310	89.9
High	35	10.1
Total	345	100.0

Our study detected a statistically significant positive correlation between spleen length and spleen volume (P < 0.05, T = 24.761, beta standardized coefficient = 0.781) (Table 4). Additionally, abdomen transverse diameter was found to be statistically significant and positively correlated with spleen volume (P < 0.05, T = 4.434, beta standardized coefficient = 0.267).

**Table 4:** Spleen volume and length relative to gender

	Spleen volume (cm3)		Spleen length (cm)	
Gender	Male	Female	Male	Female
Mean	223.5	170.27	10	8.7
Standard deviation	122.9	89.25	2.3	2.1

Associations were measured between spleen volume and other predictors such as age (P = 0.269), L1 anteroposterior (AP) diameter (P = 0.148), L1 transverse diameter (P = 0.301), abdomen AP diameter (P = 0.118), abdomen circumference (P = 0.502), and spinal canal area (P = 0.458) (Table 5). Although it appears statistically insignificant, our overall linear regression model is statistically significant (R square = 0.69, ANOVA = 83.24, P < 0.05) (Table 6).

**Table 5:** Mean and standard deviation for dependent and independent variables AP: Anteroposterior, STD: Standard deviation

	Age	Spleen length	Spleen volume	L1 AP diameter cm	L1 transverse diameter cm	L1 transverse area cm 2	Abdomen AP diameter cm	Abdomen transverse diameter cm	Abdomen circumference cm	Spinal canal area cm
Mean	51.6580	9.1802	187.2734	2.5773	3.2275	7.6177	20.9712	30.0002	99.0719	3.0757
STD	.87600	.12162	5.60141	.01740	.02047	.08918	.17574	.21203	.77325	.02996

**Table 6:** Linear regression model R Square = 0.69, ANOVA = 83.24, P-value = 0.00 Dependent variable: Spleen volume Predictors: Spinal canal area (cm), abdomen circumference (cm), age, spleen length, L1 AP diameter (cm), L1 transverse diameter (cm), abdomen transverse diameter (cm), abdomen AP diameter (cm), L1 transverse area (cm2) AP: Anteroposterior

Model		Unstandardized coefficients		Standardized coefficients	T-value	Sig.
		B	Std. Error	Beta		
	(Constant)	-310.230	57.936		-5.355	.000
	Age	-.225	.203	-.035	-1.107	.269
	Spleen length	35.961	1.452	.781	24.761	.000
	L1 AP diameter (cm)	31.412	21.649	.098	1.451	.148
	L1 transverse diameter (cm)	19.884	19.190	.073	1.036	.301
	L1 transverse area (cm2)	-9.233	6.502	-.147	-1.420	.157
	Abdomen AP diameter (cm)	-3.047	1.942	-.096	-1.569	.118
	Abdomen transverse diameter (cm)	7.062	1.593	.267	4.434	.000
	Abdomen circumference (cm)	-.309	.459	-.043	-.672	.502
	Spinal canal area (cm)	-4.288	5.777	-.023	-.742	.458

## Discussion

Splenomegaly is a notable manifestation of several diseases that may involve the liver, immune system, and hematopoietic system. Accurate noninvasive assessment of spleen volume is used in the clinical treatment of patients with these diseases [7]. 

Several studies have measured the spleen volume using different radiographic methods. In literature, the splenic volume using computed tomography was described as 201 cm3 in one study, while it was 254.94 cm3 in another study [6,9]. In the present study, the volume of the spleen was 187.27 cm3, which is lower than the volume reported in the previous studies. This could be due to variations in race, differences in measurement methods, or a smaller sample size when compared to other studies. Furthermore, the splenic volume reported by us is higher than the results of Asghar et al. [14], and Mustapha et al. [15], where they found the spleen to be 161.57 cm3 and 120 cm3, respectively.

We have measured the splenic length in the adult Sudanese population. In the studies conducted by Ehimwenma (&) Tagbo, the splenic length was found to be 10.1 cm in Nigerian females while it is found to be 11.1 in Nigerian males [16]. Moreover, Hosey et al. estimated splenic length as 9.91 cm in the American population [17]. Additionally, Çeliktas et al. found that the length of the spleen was 9.87 cm in the Turkish adult population [10]. In our data, the splenic length for the Sudanese population was found to be 10 cm in males, and 8.7 cm in females. The American, Nigerian, and Turkish populations had greater values than us. The difference in our results compared to Ehimwenma (&) Tagbo, Hosey et al., and Çeliktas et al.'s results could be due to the operator-dependent or the accuracy of the modalities used. 

Moreover, regarding gender-specific effects on splenic volume and length, we reported that the splenic volume in males and females was found to be 223.5 cm3 and 170.27 cm3, respectively, and splenic length in males and females was found to be 10 cm and 8.7 cm, respectively. This gender difference may be due to body habitus because males have a bigger body habitus than females. From a physiological perspective, the bigger the body habitus larger the blood volume, so a larger spleen is needed for filtration. Such findings could be compared to a prospective pilot study of 205 volunteers that reviewed the splenic volume obtained during an ultrasound scan of the abdomen, where the volume of the spleen was larger in males [11].

In our study, we observed spleen volume had a significant correlation with spleen length (P < 0.05). Moreover, the first lumbar vertebra anteroposterior diameter, transverse diameter, and transverse area were correlated with splenic volume with p-values (P = .148), (P = .301), and (P = .157), respectively. However, all were statistically insignificant. 

The anteroposterior abdomen diameter, transverse diameter, and abdomen circumference were 20.9 cm, 30 cm, and 99 cm, respectively. Nevertheless, the anteroposterior abdomen diameter, transverse diameter, and abdomen circumference were correlated with splenic volume with p values (P = .118), (P = .000), and (P = .502), respectively. Statistically, both abdominal anteroposterior diameter and circumference have p-values more than 0.05, and abdominal transverse diameter was correlated with splenic volume with a p-value < 0.05. We can conclude there is no relationship statistically between spleen volume and abdomen AP diameter and circumference, with significant relation between the transverse diameter and splenic volume. We did not have any previous local data for comparison. 

In addition to the limited study population, the signal-to-noise ratio of MR images and confounding factors-such as any disease that does not appear on MRI-were considered our study limitations.

## Conclusions

In conclusion, we found that the spleen volume is strongly related to spleen length and weakly related to abdominal transverse diameter. Therefore, in our case, single-slice measurements are not reliable. Furthermore, a local reference of spleen volume was established using manual segmentation and the Cavalieri method.
